# Nanoemulsions as Gene Delivery in Mucopolysaccharidosis Type I—A Mini-Review

**DOI:** 10.3390/ijms23094785

**Published:** 2022-04-26

**Authors:** Paweł Zapolnik, Antoni Pyrkosz

**Affiliations:** 1 College of Medical Sciences, University of Rzeszów, 35-959 Rzeszów, Poland; 2 Department of Clinical Genetics, College of Medical Sciences, University of Rzeszów, 35-959 Rzeszów, Poland; antoni.pyrkosz@gmail.com

**Keywords:** mucopolysaccharidosis I, genetic therapy, gene delivery, nanoparticles, nanoemulsions, review

## Abstract

Mucopolysaccharidosis type I (MPS I) is a rare monogenic disease in which glycosaminoglycans’ abnormal metabolism leads to the storage of heparan sulfate and dermatan sulfate in various tissues. It causes its damage and impairment. Patients with the severe form of MPS I usually do not live up to the age of ten. Currently, the therapy is based on multidisciplinary care and enzyme replacement therapy or hematopoietic stem cell transplantation. Applying gene therapy might benefit the MPS I patients because it overcomes the typical limitations of standard treatments. Nanoparticles, including nanoemulsions, are used more and more in medicine to deliver a particular drug to the target cells. It allows for creating a specific, efficient therapy method in MPS I and other lysosomal storage disorders. This article briefly presents the basics of nanoemulsions and discusses the current state of knowledge about their usage in mucopolysaccharidosis type I.

## 1. Introduction

### 1.1. The Basics

Mucopolysaccharidosis type I (MPS I, OMIM # 607014) is a rare monogenic disease belonging to the lysosomal storage disorders (LSDs). Severe MPS I occurs in approximately 1 in 100,000 new-borns. Attenuated MPS I is less common and occurs in about 1/500,000. Prevalence is estimated at 1/100,000, with Hurler syndrome accounting for 57% of cases, Hurler-Scheie syndrome accounting for 23% of cases, and Scheie syndrome accounting for 20% of cases. It is caused by a mutation in the *IDUA* gene, causing α-L-iduronidase deficiency (IDUA) and is inherited in an autosomal recessive way (gene *locus* 4p16.3) [1,2,3]. The most common mutation found in the European and South American populations is c.1205G>A (p.Trp402∗), resulting in the introduction of the STOP codon and premature transcription termination. It is related to the very severe phenotype of the disease [4]. The lysosomal hydrolase defect disrupts the degradation of glycosaminoglycans (GAGs), heparan sulfate (HS), and dermatan sulfate (DS), resulting in the accumulation of mucopolysaccharides in mesenchymal tissues and lipids in nervous tissue.

There are three clinical forms of this disease:

- severe—Hurler syndrome (MPS IH, OMIM #607014),
- moderate—Hureler-Scheie syndrome (MPS IH/S, OMIM #607015),
- mild—Scheie syndrome (MPS IS, OMIM #607016) [2,5].

The severe phenotype of the disease was first described by Gertrud Hurler in 1919 [6]. In 1962, Scheie et al. published a report on a milder form of the disease [7]. The moderate phenotype was characterised in 1976 by Stevenson et al. [8].

### 1.2. Clinical Features

All three forms of type I mucopolysaccharidosis differ in their clinical course and symptom severity. However, there are common features present in all forms of the disease: corneal clouding, a low bone ridge of the nose (flat base and bridge of the nose with wide, flat nostrils), thickened lips, aortic and mitral valve abnormalities, obstructive airway diseases, changes in the skeletal system known as dysostosis multiplex, and urinary excretion of dermatan sulfate and heparan sulfate. In Hurler syndrome, the first changes can already be observed in the first six months of life in the form of a mild facial dysmorphia, a macrocephaly, and limited mobility in the hip joints. There are frequent respiratory system infections. After the age of 6 months, the growth rate slows down, and a progressive intellectual disability is observed. The typical phenotype of a patient with this form of MPS I includes clinical symptoms that increase over time, such as short stature, coarse facial features, full cheeks, gingival hyperplasia, tongue enlargement, hypertrophic cardiomyopathy, joint contracture, premature overgrowth of the sagittal and frontal suture, and J-shaped sella turcica. Other symptoms observed in patients with Hurler syndrome are a tendency to hypertelorism, internal epicanthal folds, pigmentary retinal dystrophy, optic nerve compression and its atrophy, cortical visual impairment, open angle glaucoma, alveolar hypertrophy, macroglossia, short neck, kyphosis and thoracolumbar humps, underdevelopment of the odontoid process, enlargement of the shafts and deformation of the short bones, enlargement of the medial ends of the clavicles, hirsutism, hepatosplenomegaly, hernias (inguinal, umbilical), hip dislocation, tracheal stenosis, spinal cord and cranial nerves compression, and deafness [5]. Usually, breathing problems or heart failure lead to death in childhood. Hurler patients rarely live to the age of 10. In the moderate form of the disease—Hurler-Scheie syndrome—symptoms occur between 3 and 8 years of age, and patients usually live up to 20 years. Symptoms of the disease may be similar to the severe form, but the intellectual disability is mild, and the image of the coarse face is not as intense. Patients with Scheie syndrome may have normal intellectual development, and the first symptoms are not found until the second and third decades of life. Patients may develop eye damage or skeletal abnormalities limiting physical ability, while the life expectancy does not differ from the average value for the population [5,9,10]. The sporadic abnormalities seen in MPS I patients include hydrocephalus, probably due to thickening of the cerebral meninges, arachnoid cysts, hydrocoele, nephritic syndrome, carpal tunnel syndrome, bent thumb, and hypotrophy of the condylar process of the mandible [5].

### 1.3. Diagnosis and Management

The diagnosis confirmation is based on the presence of increased levels of dermatan sulphate and heparan sulphate in the urine and the absence of α-L-iduronidase activity in peripheral blood leukocytes or skin fibroblasts. Prenatal diagnosis is possible by assessing the enzyme activity in the cells of the amniotic fluid and detecting the mutation in the material from amniocentesis or chorionic villus sampling [5]. Molecular methods are used to determine the mutation in the *IDUA* gene and evaluate its significance [10].

The therapeutic possibilities of MPS I are limited and rely on multidisciplinary care, but scientists are still attempting to apply new curative methods. Thanks the discovery of the phenomenon of cross-correction, i.e., the secretion of the enzyme by some cells and its uptake by others via the mannose-6-phosphate receptor, the work on obtaining therapeutic agents was accelerated [11,12]. Currently, an enzyme replacement therapy (ERT) using recombinant human α-L-iduronidase administered intravenously is mainly used. It effectively inhibits liver enlargement and improves mobility in joints, respiratory capacity, and heart function. However, the enzyme cannot cross the blood–brain barrier (BBB) by itself, so this method does not correct the function of the central nervous system (CNS). Another available treatment is hematopoietic stem cell (HSCT) transplantation, which has been successful when applied before the age of 24 months. The transplantation generally increases survival by 67%. This procedure prevents the coarsening of facial features and enlargement of the liver and spleen and improves hearing, respiratory and heart function. Children who have undergone transplantation before the age of 24 months and who score 70 or more points on the Mental Developmental Index scale may show beneficial neural and behavioural development. Despite this, progression of valvular insufficiency, skeletal abnormalities, and corneal clouding are often found. [5,13,14]. However, this procedure carries a risk of complications due to the immunosuppressive therapy and the graft versus host disease (GvHD).

Researchers attempt to overcome these difficulties by using new techniques, including gene therapy. Gene therapy refers to the use of nucleic acids to treat/modify the course of disease in humans. Due to the used carrier of the reconstructed genetic information, it can be divided into virus-based and non-viral gene therapy. In the first case, the correct gene or genome editing system is delivered by a virus. The first ever attempt at gene therapy took place almost 30 years ago in severe combined immunodeficiency due to adenosine deaminase deficiency [15]. The first vectors used in this treatment method were retroviruses and lentiviruses. However, they were characterised by limitations (random integration with the genome and immunogenicity) that reduced their usefulness. Nowadays, there are usually used adenovirus-associated viruses (AAVs), ensuring a broad and stable gene expression. Still, it is associated with the risk of immunogenicity and mutagenesis. An alternative is the use of non-viral vectors, e. g., nanoparticles or the Sleeping Beauty transposon system [16,17,18]. These methods do not have the limitations of viruses and are being developed more and more. In the following part of the review, we would like to present the current possibilities of gene therapy using nanoemulsions in mucopolysaccharidosis type I.

## 2. Nonviral Gene Therapy with Nanoemulsions

### 2.1. The Basics

Colloidal carriers, such as liposomes and solid or polymeric nanoparticles, have increasingly been used as non-viral vectors in gene therapy. Among these, cationic liposomes were the first to be more widely used [19]. The first use of a cationic emulsion as a vector for gene delivery in vivo that took place in 1997. Hara et al. [20] injected an emulsion composed of castor oil and cholesteryl carboxyamidoethylenedimethylamine containing a plasmid with the gene into the portal vein of a mouse. They achieved increased gene expression in the liver.

Nanoemulsions are thermodynamically stable colloidal dispersions consisting of the molecule aggregation of two phases: oil in water (O/W) or water in oil (W/O). Sometimes multiple nanoemulsions (W/O/W) are also used [21]. They are made of oils, surface-active compounds, and water, and the mean size of a single lipid ‘droplet’ is usually less than 500 nm. For example, the oil components used are short-chain triglycerides, medium-chain triglycerides, sesame oil, cottonseed oil, safflower oil, coconut oil, ricebran oil, soybean oil, or squalene. Surface-active compounds reduce the interfacial tension and avoid droplet aggregation. The wide-spread surfactants used are castor oil and cholesteryl carboxyamidoethylenedimethylamine; stearylamine; lecithin (phosphatidylcholine); sorbitan monolaurate; polyoxyethlene sorbitan monolaurate; sodium dodecyl sulfate; 1,2-dioleoyl-snglycero-3-trimethylammonium-propane (DOTAP); casein, β-lactoglobulin; and polysaccharides [21,22]. To support the function of surfactants, co-surfactants that stabilise the phase border can be used. These include poly (ethylene glycol), ethanol, glycerine, ethylene glycol, and propanol. Other essential ingredients added during nanoemulsion development are preservatives and antioxidants to prevent oxidation. These include, e.g., ascorbic acid and ascorbic acid esters, sodium bisulfite, thiourea, sodium formaldehyde sulfoxylate, butyl hydroxytoluene, citraconic acid, phosphoric acid, citric acid, and tartaric acid [21].

Nanoemulsions show good bioavailability, and, most importantly, they can cross the blood–brain barrier (BBB) [22]. The use of various modifications enables the stabilisation of nanoemulsions against multiple factors in the in vivo environment. For example, enriching the nanoemulsion with omega-3 polyunsaturated fatty acids (PUFA) will ensure easier passage through the BBB and faster drug delivery to target cells. Using the 1,2-dioleoyl-sn-glycero-3-trimethylammonium propane (DOTAP) compound will provide a positive surface charge so that the ‘droplets’ will not stick together, and their desired size will be achieved [22,23,24]. The use of fatty acids with amino acid modification, e.g., lauroyl-arginine methyl ester, increases the bioavailability of the agent and reduces its toxicity [19]. Another compound that modifies the biophysical characteristics of nanoemulsions is the polysaccharide complex chitosan. The positive charges of chitosan interact with negatively charged groups of nucleic acids. In addition, it provides good adhesion to the cell, which allows greater efficiency of gene delivery to the target site. An additional modification is the use of dyes, which would enable the determination of the location of nanoemulsion particles in tissues. An example of a fluorescent agent used is IR-780 cyanine, which belongs to the group of polymethine cyanine dyes (indocyanines) [25]. Furthermore, other compounds can also be added to nanoemulsions, such as the MF-59 adjuvant used in vaccines. This is a safe and tested agent that was used by Brito et al. [26] in a study evaluating the possibility of using cationic nanoemulsions as a delivery system for mRNA vaccines.

Nanoemulsions may be produced by high-energy methods or low-energy methods. The first method mentioned above involves generating destructive forces to reduce the size of the droplets. It includes microfluidisers, ultrasonicators and piston-gap homogenisers. Low-energy methods rely on the cumulative self-organising of polymers. The essence of these methods is using the energy of the system to produce very small particles. These may include spontaneous emulsification and the phase inversion method. Nanoemulsions can be administered in various forms (e.g., liquids or aerosols) by oral, intravenous, intranasal, intraocular, or topical routes [21].

Cationic nanoemulsions are excellent compounds for delivering nucleic acids to the human body because they form nanocomplexes with negatively charged DNA, thanks to which DNA is protected against enzymatic degradation in body fluids. In addition, they are stable during transport in the blood and are also rarely recognised by the immune system cells. The effectiveness of combining nanoemulsions with nucleic acid and its detachment depends on the ratio of charges (+/–) between cationic lipids and phosphate groups of nucleic acids. Cationic lipid particles are currently the most widely studied non-viral vectors [27,28]. 

### 2.2. Gene Therapy

Schuh et al. [18] conducted a study evaluating lipid nanoemulsions as a means of delivering donor oligonucleotide to cells using the clustered regularly interspaced short palindromic repeat/Cas9 (CRISPR/Cas9) genome editing technique. The authors used in cell culture human fibroblasts collected from a patient with Hurler syndrome with the p.Trp402∗/p.Trp402∗ genotype. The nanoemulsion particles consisted of an oil core and cationic lipids attached to the phosphate groups of nucleic acids via electrostatic interactions. Researchers used two techniques to obtain two forms of nanoemulsions. In the first one, DNA adsorption was carried out on the surface of nanoemulsion particles, while in the second, DNA complexes with 1,2-dioleoyl-sn-glycero-3-trimethylammonium propane (DOTAP) were implemented in the oily core. The diameter of the nanoemulsion droplets was 175 to 214 nm. Assessment of genetic material transfer efficiency to cells was performed on days 2, 15, and 30 after transfection. The addition of nanoemulsions to the cell culture increased the activity of α-l-iduronidase to an average level of 2–4% of the normal activity. Analysis of fibroblasts under confocal microscopy revealed a reduction in the accumulation of lysosomes in the cells, subsequently confirmed by flow cytometry. Additionally, the authors assessed the stability of the complexes by incubating them in cell culture. They showed that the DNA/DOTAP complexes inside the ‘droplet’ have twice as long a lifetime as the DNA adsorbed on the molecule’s surface (48 h compared to 24 h) [18]. The study results presented above are promising, but a successful in vitro experiment does not necessarily lead to similar results in vivo. It is essential to trace the results of the obtained studies on model organisms.

In a study by Fraga et al. [29], the authors assessed the behaviour of nanoemulsions in the mouse MPS I model. They used lipid nanoemulsions as a carrier for plasmid DNA for encapsulation and a pegylated nanoemulsion for adsorption. The diameter of the emulsion droplets was approximately 200 nm. The laboratory model was 6-month-old *IDUA knockout* mice. The animals were sacrificed 48 h after intravenous administration of the solution, and the authors collected for further examination following tissues: lungs, kidneys, liver, and spleen. The activity of the protein product of the *IDUA* gene was detected in all tissues collected for analysis. Additionally, the expression level of the gene itself was more significant than in the control mouse population with MPS I, which was not given nanoemulsions. Additionally, the authors have shown that pegylation of nanoemulsions makes it more stable [29]. In the cited study, the use of nanoemulsions as a carrier for plasmids coding for normal α-L-iduronidase has been shown to be effective in a mouse model. This enables a broader evaluation perspective than cell culture.

In another work by Fraga et al. [30], researchers assessed the effectiveness of nanoemulsion transfection with a plasmid containing the *IDUA* gene depending on the dose and time of drug action. They performed an analysis using a similar cationic nanoemulsion to the one in the previously mentioned study [29], using DNA adsorption and encapsulation on the MPS I mouse model. Mice were administered 30 or 60 µg of nanoemulsion, and then the *IDUA* gene expression and the enzyme activity in the tissues were assessed after 2 or 7 days. The authors demonstrated increased activity of the *IDUA* in the lungs and liver, with the effect being better after using a higher dose and after seven days of observation. Histopathological examination did not show any increased signs of necrosis, apoptosis or inflammation, and the only side effect of the therapy was minor hydropic degeneration in hepatocytes [30]. The above study shows that using nanoemulsions as a carrier of nucleic acids in gene therapy may have a dose and time-dependent effect. Therefore, it is possible to determine the right amount of the agent that can give the most optimal therapeutic effect.

Fraga et al. [31] conducted further gene therapy studies on a mouse model of mucopolysaccharidosis type I. They assessed the efficacy and safety of repeated doses of pegylated nanoemulsion with the *IDUA* plasmid. Repeated administration of the same doses of nanoemulsion to mice resulted in increased expression of the *IDUA* gene and increased enzyme activity in tissues concerning a single amount of the preparation. Histological analysis additionally showed a reduced concentration of GAGs and a reduced presence of CD68+ macrophage cells compared to untreated MPS I mice [31]. This recently published study provides further information on the safety of nanoemulsion gene therapy so far in an animal model, but obtaining such data may accelerate further efforts to implement this therapy in humans (introducing a clinical trial).

One of the common health problems in patients with MPS I is skeletal abnormalities described as multiplex dysostosis. Contractures and stiffness of the joints and limitation of their mobility lead to numerous complications. Bidone et al. [32] conducted a nanoemulsion study with an adsorbed plasmid containing the *IDUA* gene. The authors assessed the efficacy of transfection in a culture of fibroblast-like synoviocytes (FLS) and after intra-articular administration to MPS I mice. They observed an increased enzyme activity in cell culture. On the other hand, intra-articular administration of the preparation resulted in complex transfection of synoviocytes and increased alpha-L-iduronidase activity. This intervention was local, as there was no significant increase in enzyme activity in other tissues [32]. This study shows that the ongoing trials of the therapeutic application of nanoemulsions bring effects on model organisms also in terms of local application.

The storage of heparan sulfate and dermatan sulfate in the central nervous system and abnormal intellectual development is one of the most severe complications of impaired glycosaminoglycans metabolism. However, the authors did not determine enzyme activity or gene expression in the CNS in the above trials. Improving neurological functions, including cognitive function, is one of the main challenges faced by researchers dealing with MPS I therapy, as it is associated with a direct improvement in the quality of life of these patients. Schuh et al. [33] applied a complex of nanoemulsions and a plasmid encoding *IDUA* intranasally in MPS I mice. The gene expression and the enzyme activity have been demonstrated in the mice’s brains, additionally confirming their presence in the kidneys and spleen. Histopathological examination also showed no signs of inflammation in the central nervous system. The above results are encouraging and could form the basis for the potential treatment of CNS dysfunction in patients with MPS I. A summary of the usage of nanoemulsions as a gene therapy method is presented in Table 1.

## 3. Challenges and Perspective

Gene therapy is a promising and continuously developed method of treating monogenic diseases. It was first used in humans in the early 1990s in severe combined immunodeficiency associated with adenosine deaminase deficiency [15]. Since then, a lot of progress has been made in this area. Viral vectors have significant limitations related to immunogenicity and the mutagenesis risk (retroviruses, lentiviruses). Antibodies are produced to a lesser extent against AAVs, making them the most optimal viral vector nowadays [34]. In addition to the virus as a vector, elements of the genome editing systems may also be immunogenic [35]. This effect is minor with nanoemulsions, and it is their main advantage. In addition, they are easy to obtain; they can be combined with various molecules; and, with appropriate modifications, they can easily cross biological barriers.

Despite all their advantages, nanoemulsions have limitations, and there are still many challenges for researchers to overcome. First, all available studies as of today have been performed on cell lines or laboratory organisms. Even the best model of mucopolysaccharidosis type I will not reflect the actual conditions in the human body. The intravenous solution itself may prove less stable in vivo, and plasma enzymes may degrade the conjugated DNA. The true expression of the gene in target cells is also difficult to predict.

Due to the lack of data on the use of nanoemulsions in mucopolysaccharidosis type I in humans, further studies, including randomised clinical trials, are needed to assess the safety and effectiveness of such therapy. It is also necessary to analyse possible interactions in the human body and identify factors influencing the efficacy of in vivo transfection.

**Figure 1:**
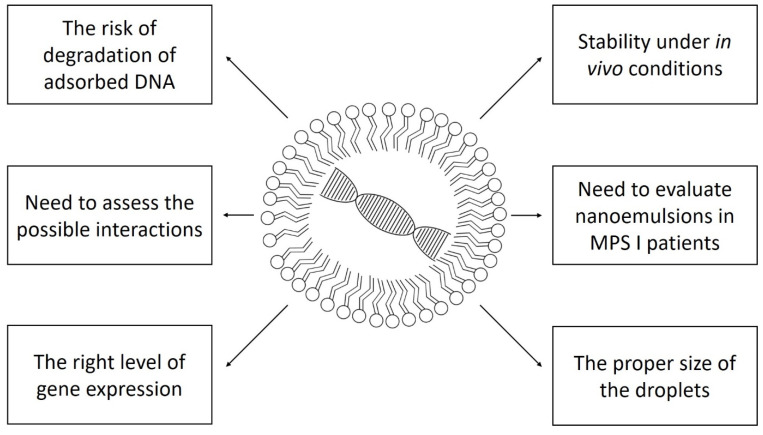
The most important problems standing in the way to the widespread use of nanoemulsion gene therapy in mucopolysaccharidosis type I.

Clinical trials with gene therapy are conducted in patients with mucopolysaccharidosis type I. However, they are based on viral vectors or autologous transplantation of modified CD34+ hematopoietic stem cells [36]. Nanoemulsions used with various drugs are applied in clinical trials in neoplastic, ophthalmological, dermatological, and haematological diseases. So far, there are no registered clinical studies using nanoemulsions in storage diseases, including mucopolysaccharidoses [37]. Figure 1 shows schematically the problems that must be solved to safely and effectively use nanoemulsion gene therapy in humans.

## 4. Conclusions

Mucopolysaccharidosis type I, as a monogenic disease, is the optimal target of gene therapy. So far, therapeutic possibilities are limited to multidisciplinary care and enzyme replacement therapy or hematopoietic stem cell transplantation. Clinical trials with the use of gene therapy based on viral vectors are underway. Despite numerous studies in cell cultures and animal models, no attempts have been made to use nanoemulsions as a vector for genetic transfer in MPS I patients. The further development of molecular biology methods and the improvement of non-viral carriers will probably contribute to the application of this type of gene therapy also in humans. More studies are needed to assess the safety and efficacy of these agents in animals. Additionally, the first clinical studies in patients with Hurler syndrome are necessary. The combined use of viral and non-viral vectors could also be considered to increase the current knowledge. Possibly, the initiation of an additional branch of research on model organisms or clinical trials in patients with Hurler syndrome would provide new information on the effectiveness of different ways of delivering the gene to the target cells. Such studies could compare the use of other vectors and their routes of delivery into the body. Such activities could undoubtedly accelerate the achievement of an effective method of treatment, including the improvement of the central nervous system functions. This will directly translate into the development and quality of life of the patients.

## Figures and Tables

**Table 1:** The use of nanoemulsions in preclinical trials in mucopolysaccharidosis type I.

Features	Nanoemulsions Gene Transfer
Main benefits	No risk of mutagenesis Easy to produceEasy to modify Ease of incorporating various therapeutic agents into nanoemulsions
Main limitations	Risk of lower stability in relation to other nanoparticles The ‘droplets’ may be too large Risk of uncertain gene expression Immunogenicity
Routes of administration in model organisms	Intravenous [23] Intraarticular [26] Intranasal [27]

## Data Availability

No new data were created or analyzed in this study. Data sharing is not applicable to this article.

## Abbreviations

BBB	Blood–brain barrier
CNS	Central nervous system
CRISPR	Clustered regularly interspaced short palindromic repeat
DOTAP	1,2-dioleoyl-*sn*-glycero-3-trimethylammonium propane
DS	Dermatan sulphate
ERT	Enzyme replacement therapy
FLS	Fibroblast-like synoviocytes
GAGs	Glycosaminoglycans
GvHD	Graft versus host disease
HS	Heparan sulphate
HSCT	Hematopoietic stem cell transplantation
IDUA	Alpha-L-iduronidase
LSDs	Lysosomal storage disorders
MPS I	Mucopolysaccharidosis type I
PUFA	Omega-3 polyunsaturated fatty acids
Trp	Tryptophan
